# *MEIS1* variant as a determinant of autonomic imbalance in Restless Legs Syndrome

**DOI:** 10.1038/srep46620

**Published:** 2017-04-20

**Authors:** Jérôme Thireau, Charlotte Farah, Nicolas Molinari, Fabrice Bouilloux, Lucas Torreilles, Juliane Winkelmann, Sabine Scholz, Sylvain Richard, Yves Dauvilliers, Frédéric Marmigère

**Affiliations:** 1 INSERM U1046, UMR CNRS 9214, University of Montpellier, 371, avenue du doyen Gaston Giraud, 34295 Montpellier Cedex 05, France; 2 Institut Montpelliérain Alexander Grothendieck, CNRS, University of Montpellier, Department of Statistics, Montpellier, France; 3 INSERM U1051, Institute for Neurosciences of Montpellier (INM), 80 rue Augustin Fliche, 34091 Montpellier, France; 4 Institute for Neurogenomic, Helmholtz Zentrum München, Neuherberg; Neurologische Klinik und Poliklinik Munich, Germany; 5 Sleep Unit, Department of Neurology, Gui-de-Chauliac hospital, INSERM U1061, Montpellier, France

## Abstract

Restless Legs Syndrome (RLS) is a genetically complex neurological disorder in which overlapping genetic risk factors may contribute to the diversity and heterogeneity of the symptoms. The main goal of the study was to investigate, through analysis of heart rate variability (HRV), whether in RLS patients the *MEIS1* polymorphism at risk influences the sympathovagal regulation in different sleep stages. Sixty-four RLS patients with periodic leg movement index above 15 per hour, and 38 controls underwent one night of video-polysomnographic recording. HRV in the frequency- and time- domains was analyzed during nighttime sleep. All RLS patients were genotyped, and homozygotes for rs2300478 in the *MEIS1* locus were used for further analysis. Comparison of the sympathovagal pattern of RLS patients to control subjects did not show significant differences after adjustments for confounding factors in frequency-domain analyses, but showed an increased variability during N2 and N3 stages in time-domain analyses in RLS patients. Sorting of RLS patients according to *MEIS1* polymorphism reconfirmed the association between *MEIS1* and PLMS, and showed a significant increased sympathovagal balance during N3 stage in those homozygotes for the risk allele. RLS patients should be considered differently depending on *MEIS1* genotype, some being potentially at risk for cardiovascular disorders.

Restless Legs Syndrome (RLS), also known as Willis-Ekbom disease, is a genetically complex neurological disorder in which overlapping genetic risk factors contribute to the diversity and heterogeneity of the symptoms^1^,^2^. Genome-wide association studies have evidenced common genetic variants on at least six genomic regions which, according to their odd ratios, moderately influence RLS symptoms^1^,^2^. Among them, *MEIS1*, a gene coding for a transcription factor of the TALE (Three Amino acid Loop Extension) Homeodomain family, is the largest risk factor identified so far^3^,^4^,^5^. In various species, *Meis1* has been implicated in the development of several peripheral organs and nervous system structures including the striatum, the retina, the forebrain, the cerebellum and sympathetic neurons^6^,^7^,^8^,^9^,^10^. The SNPs (Single-Nucleotide Polymorphisms) identified in the eighth intron of the *MEIS1* gene suggest that deregulations of mRNA transcription and/or processing participate in the etiology of RLS. Accordingly, a decrease in MEIS1 mRNA and protein has been detected in RLS patients carrying these SNPs^11^. In addition, the SNP rs12469063 is located in a conserved genomic region across species that serves as a neuronal enhancer for the *Meis1* gene, and transgenic reporter mice carrying this risk allele exhibit a reduced enhancer activity, providing the first evidence for a direct link between this SNP and the neuronal levels of Meis1 protein^12^.

The comorbidity between RLS and cardiovascular diseases has been largely investigated with conflicting results explained in part by individual heterogeneity among the populations investigated^13^,^14^,^15^,^16^. Moreover, a dysfunction or deregulation of the sympathetic nervous system (SNS) leading to tachycardia and high blood pressure has been hypothesized^14^,^16^,^17^,^18^,^19^. About 60–80% of the patients also present periodic limb movements during sleep (PLMS), a pattern that is believed to increase cardiovascular risk, congestive heart failure, atrial fibrillation and coronary heart disease^18^,^20^,^21^. RLS patients with PLMS may be thus at risk for heart diseases and hypertension^18^. PLMS episodes occur simultaneously with activation of the SNS as revealed by Heart Rate Variability (HRV) analysis and a rise in blood pressure^22^,^23^,^24^,^25^. In the *MEIS1* locus, rs2300478 and rs12469063 were found to be significantly more associated with PLMS than RLS suggesting a primary function of this gene in the generation of PLMS independently of RLS symptoms^26^,^27^.

The goals of the study were to: 1/compare RLS patients and healthy control subjects through Heart Rate Variability (HRV) analysis of ECG acquired during polysomnographic (PSG) recording; and 2/assess if the *MEIS1* SNP rs2300478 influences the sympathovagal regulation according to sleep stages in RLS patients.

## Results

**Figure 1:**
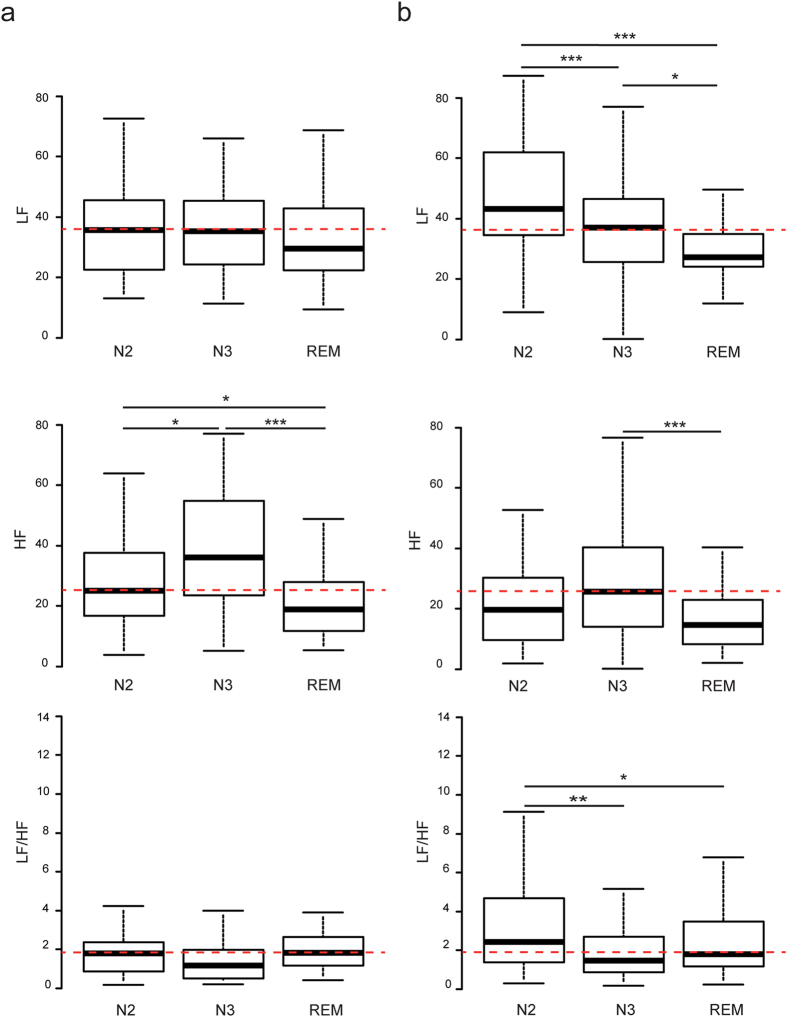
HRV analysis in the frequency domain in RLS patients and control subjects. Box-whisker plots showing LF, HF and LF/HF in control subjects (**a**) and in RLS patients (**b**) during N2, N3 and REM sleep stages. Data are represented as lower quartile, median and upper quartile (boxes), and minimum and maximum ranges (whiskers). Red dashed lines indicate the median values for LF, HF and LF/HF in control patients during N2 (A and B). *p* = p value following Student’s or Wilcoxon’s tests; *p ≤ 0.05; **p ≤ 0.01; ***p ≤ 0.005.

From frequency domain analysis, in RLS patients, the LF (Low Frequency power) band reflecting in part the sympathetic component of autonomic nervous system varied considerably during the different sleep stages with higher LF_N2_ than LF_N3_ and LF_REM_ (*p* = 0.005 and 1.10^−4^ respectively), and higher LF_N3_ than LF_REM_ (*p* = 0.02) (Fig. 1b). HF (High Frequency power) values showed a less pronounced variation with sleep stages but HF_N3_ and HF_REM_ were statistically different (*p* = 0.005). The increased in LF band without major variation of HF band resulted in a significantly higher (LF/HF)_N2_ than (LF/HF)_N3_ and (LF/HF)_REM_ (*p* = 0.002 and 0.037 respectively). In contrast, control subjects had stable LF and LF/HF values throughout sleep stages but with a dominant HF activity during N2 (Stage 2 of Non-Rapid Eye Movement (NREM) sleep) and N3 (Stage 3 of NREM sleep) stages with significantly higher HF_N2_ and HF_N3_ than HF_REM_ (*p* = 0.04 and 1.10^−4^ respectively), and higher HF_N3_ than HF_N2_ (*p* = 0.02) (Fig. 1a). From time domain analysis, RRmean values significantly changed in control subjects during the different sleep stages with RRmean_N3_ being lower than RRmean_N2_ and RRmean_REM_ (*p* = 0.001 and *p* = 0.05 respectively). SDNN_N3_ (Standard Deviation of all normal RR intervals) was lower than SDNN_N2_ and SDNN_REM_ (p = 5.10^−5^ and 0.0003 respectively), and RMSSD_N3_ (Root Mean Square of Successive Differences) was lower than RMSSD_N2_ (p = 0.009) (Supplementary Fig. S1a and Table S1). From Poincare plot geometry analysis in control subjects, SD1_N3_ (Standard Deviation 1) was lower than SD1_N2_ (p = 0.008), and SD2_N3_ (Standard Deviation 2) was lower than SD2_N2_ and SD2_REM_ (p = 2.10^−5^ and 3.10^−6^ respectively) (Supplementary Fig. S2a and Table S2). Altogether, these time domain data indicate than in control subjects, cardiac rhythm was more stable during N3 stage than during N2 and REM stages. In contrast, Mean RR values did not significantly vary in RLS patients while SDNN fluctuated between sleep stages with a less regular rhythm in N2 than in N3 and REM (Rapid Eye Movement) stages as indicated by higher SDNN_N2_ than SDNN_N3_ (*p* = 0.02) and SDNN_REM_ (*p* = 0.01) (Supplementary Fig. S1b and Table S1). This observation was also confirmed by RMSSD variations, with RMSSD_N2_ being higher than RMSSD_REM_ (*p* = 0.003). Results from Poincare plot geometry analysis also underlined this increase in total variability during N2 stage in RLS patients, with both short- (SD1) and long-term (SD2) variations of RR during N2 stage being significantly higher than during REM stage (*p* = 0.003 and *p* = 0.03 respectively). Contrary to control subject, SD1_N3_ and SD2_N3_ in RLS patients were not significantly different than during N2 and REM stages (Supplementary Fig. S2 and Table S2).

Compared with controls, patients with primary RLS were older and more frequently men, without significant differences for BMI (Body Mass Index) and ferritin levels (Table 1). As expected, a lower total sleep time and sleep efficiency together with a higher wake up time after sleep onset, PLMS_REM_ and PLMS_NREM_ indexes were found in RLS patients. Between-group comparison showed a different LF profile according to sleep stages with increased LF_N2_ and (LF/HF)_N2_ and decreased HF_N3_ in RLS patients (*p* = 0.002, 0.004 and 0.015 respectively) (Table 2). However, we found no significant differences after adjustment for age and sex (Table 2), and other potential confounders such as PLMS (data not shown). Using time-domain analysis, SDNN and RMSSD revealed that during both N2 and N3 stages, RLS patients present a higher variability than control subjects (*p*_*a*_ = 0.0003 and 0.003 for SDNN during N2 and N3 respectively, and *p*_*a*_ = 2.10^−4^ and 0.0026 for RMSSD during N2 and N3 respectively) (Supplementary Fig. S1 and Table S1). In RLS patients, SD1_N2_, SD1_N3,_ SD2_N2_ and SD2_N3_ were all significantly increased in RLS patients compared to control subjects (*p*_*a*_ = 2.10^−4^ and 4.10^−3^ for SD1 during N2 and N3 respectively, and *p*_*a*_ = 8.10^−3^ and 0.019 for SD2 during N2 and N3 respectively) (Supplementary Fig. S2a,b and Table S2). Altogether, time domain and Poincare plot geometry analyses confirmed the higher variability during N2 and N3 stages in RLS patients compared to control subjects. RLS patients also exhibited a higher variability during REM as seen by SDNN (*p*_*a*_ = 0.03), RMSSD (*p*_*a*_ = 0.017) and SD1 (*p*_*a*_ = 0.017) (Supplementary Fig. S2a,b and Table S2).

**Figure 2:**
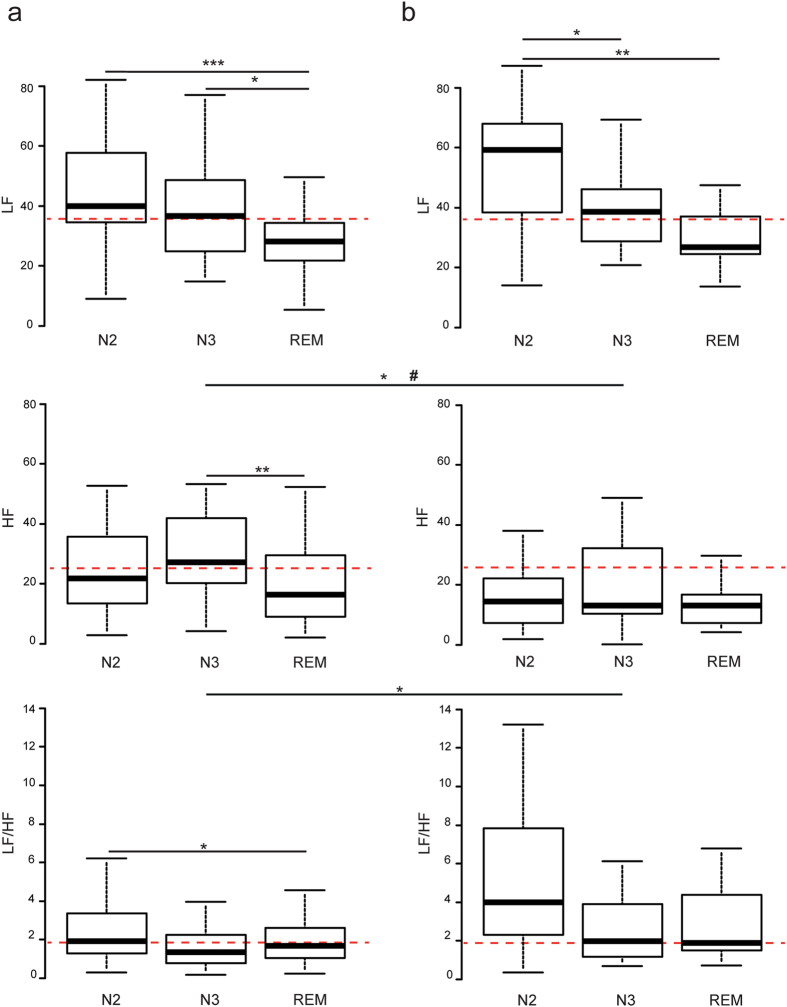
HRV analysis in the frequency domain in RLS patient as a function of the *MEIS1* locus. Box-whisker plots showing LF, HF and LF/HF in RLS TT (**a**) and RLS GG (**b**) patients during N2, N3 and REM sleep stages. Red dashed lines indicate the median values for LF, HF and LF/HF in control patients during N2 (**a** and **b**). Data are represented as lower quartile, median and upper quartile (boxes), and minimum and maximum ranges (whiskers). *p ≤ 0.05; **p ≤ 0.01; ***p ≤ 0.005 following Student’s or Wilcoxon’s tests. ^#^*p*_*a*_ ≤ 0.05 following statistical test adjusted for PLMS.

No demographic, clinical and PSG significant differences were found between patients with *MEIS1* SNP rs2300478 TT and GG, except for a higher PLMS_NREM_ index in sleep in patients GG (Table 1). HRV analysis in the frequency domain indicated that in the RLS GG group, LF_N2_ was significantly higher than LF_N3_ (*p* = 0.019) and LF_REM_ (*p* = 0.003). In the RLS TT group, LF_N2_ and LF_N3_ were significantly higher than LF_REM_ (*p* = 1.10^−4^ and 0.011 respectively). Between-group comparison showed that patients GG had a reduced HF power suggesting lower parasympathetic activity during N3 stage in crude analysis (*p* = 0.02) and after adjustment for PLMS (*p* = 0.03) (Fig. 2). No between-group significant differences were found in LF and LF/HF in any of the sleep stages after adjustment for age, gender and PLMS (Table 2). However, the sympatho-vagal LF/HF ratio in the RLS GG group was higher than in the TT group during N3 stage in crude analysis, a finding confirmed by time domain analysis. In the GG group, RRmean did not vary during the different sleep stages, whereas in the TT group, RRmean_N2_ was higher than RRmean_REM_ (*p* = 0.02). Poincare plot analysis indicated a higher variability during N2 stage than during REM stage in the TT group as seen by a higher SD1_N2_ than SD1_REM_ (*p* = 0.03), whereas in the GG group, variability during N2 was higher than during both N3 and REM stages with higher SD1_N2_ than SD1_N3_ and SD1_REM_ (*p* = 0.03 and 0.04 respectively). Accordingly, in time domain analysis, RMSSD_N2_ was higher than RMSSD_REM_ in the TT group (*p* = 0.03), whereas in the GG group, RMSSD_N2_ was higher than both RMSSD_N3_ and RMSSD_REM_ (*p* = 0.03 and 0.04 respectively) (Supplementary Fig. S2c,d and Table S2). Finally, comparison of the TT and GG groups in time domain and Poincare plot geometry analyses showed a higher variability with a more complex pattern in the RLS GG group than in the TT group, with higher SDNN_N2_, RMSSD_N2_, SD1_N2_ and SD2_N2_ in the GG group (*p* = 0.025, 0.02, 0.021 and 0.036 respectively) (Supplementary Figs S1c,d and S2c,d and Tables S1 and S2). After adjustment for PLMS, only SDNN_N2_ and SD2_N2_ remained significantly higher in the GG group than in the TT group (*p*_*a*_ = 0.024 and 0.028 respectively). No significant differences were found between the GG group and the TT group during N3 and REM after adjustment for PLMS in time domain and Poincare plot geometry analyses.

## Discussion

This study showed that *MEIS1* SNP rs2300478 at risk for RLS influence the sleep stage-dependent sympathovagal balance in RLS patients. HRV analysis in the frequency domain revealed a reduction in the parasympathetic activity during slow-wave N3 stage in those homozygotes for the risk allele, whereas time domain and Poincare plot geometry parameters indicated an increased HRV during N2 and N3 stage in RLS patients-independently of *MEIS1* genotype. In contrast, frequency domain parameters were not significantly different between patients with RLS and controls after adjustments for confounding factors.

Control subjects presented the expected pattern of sympathovagal balance during the different stages of sleep with stable LF values throughout sleep stages but a dominant parasympathetic activity during NREM sleep particularly in deep N3 sleep^28^,^29^,^30^,^31^. These results confirmed that in normal subjects, the sympathovagal balance during sleep is mostly influenced by the parasympathetic activity. In RLS patients, although the parasympathetic nervous system followed a similar pattern of activity than in control subjects, the overall sympathovagal balance tended to shift in favor of a predominant sympathetic activation, associated with reduced vagal influences, especially during N2 and N3 stages. However, we found no further significant changes between groups in frequency domain analysis when considering potential confounding factors such as age and gender. The most striking difference we found concerned the lack of parasympathetic activation during slow-wave sleep in RLS patients as a function of *MEIS1* genotype. Indeed, only the RLS GG group did not increase HF during N3 although the LF band was identical in the TT group. We also confirmed the association between *MEIS1* and PLMS with higher PLMS index in RLS patients GG for rs2300478^26^,^27^. The reduced HF_N3_ activity found in these patients persists after adjustment for PLMS. A lack of parasympathetic responsiveness has been suggested in N2 stage during PLMS in children^32^ and in RLS patients during wakefulness by measuring the Valsalva ratio^19^. A weak and non-significant reduction of HF has also previously been reported during PLMS in N2 sleep in RLS patients^25^. The increased sympathetic activity leading to shortening of RR interval (Inter-beat interval) and a rise in systemic blood pressure preceding the onset of PLMS has previously been reported^17^,^22^,^23^,^24^,^25^,^33^,^34^,^35^. In RLS patients, the changes in HRV accompanying the PLMS are also more pronounced than in healthy patients^35^.

Time domain and Poincare plot geometry analyses indicated that in control subjects, N3 stage exhibited less variability and complexity than other sleep stages as seen by RRmean, SDNN and SD2, and to some extend by RMSSD and SD1. Although the clinical and physiological interpretation of increased variability and complexity in time domain and Poincare plot geometry analyses remains elusive^36^, and taking in account that these analyses should be performed on ECG recording longer than in the present study^37^, the lack of significant changes of RRmean, RMSSD, SD1 and SD2 during N3 in RLS patients compared to N2 and REM stages may reflect the low sympathovagal balance suggested by analyses in the frequency domain. Future analyses should be performed using new nonlinear/complexity methods^38^ to confirm and refine our conclusions and tempt to establish a clinical significance. Finally, the increased variability and complexity during N2 in RLS patients is consistent with the non-significant increase in the LF band.

Whereas increased variability during wake is commonly associated to a better prognosis in various situations such as heart failure^39^ or after myocardial infarction^40^, the consequence on the increased variability and complexity on short ECG sequences during N2 and in particular during N3 in RLS patients observed in time domain analysis can only be speculative. During N3, in normal subject, the autonomic balance driven by a parasympathetic dominance is part of a complex physiological response that has been referred as the cardiovascular holiday^41^. Thus, the increased variability and complexity seen by our time domain and Poincare plot geometry analyses during N3 stage in RLS patients, and reflecting a less stable cardiac rhythm, might be considered in the evaluation of the cardiovascular risk in RLS patients.

Our study highlights a potent sympatho-vagal balance resulting from a low parasympathetic modulation in RLS patients in ECG periods free of PLMS, and so, out of baroreflex adjustment of heart rate due to leg movement. This scenario suggests that these autonomic variations might play a primary role in the generation of the PLMS^17^. Accordingly, it has been proposed that a generator located at spinal level modulated by descending dopamine inhibitory pathways is involved in the generation of PLMS^42^,^43^. Experimental evidences converged to promote dopaminergic neurons of the hypothalamic A11 nucleus as a strong candidate likely involved in the promotion of PLMS and sympathetic activity^44^. Meis1 is widely expressed in the nervous system^7^,^9^,^10^ and recent Magnetic Resonance Imaging studies of whole brain functional connectivity in RLS patients demonstrated alterations in cortical and subcortical network, suggesting potential involvement of cortical, subcortical, spinal and peripheral nervous areas^45^. We recently showed that during mouse sympathetic neurons development, Meis1 serves as a dedicated maintenance factor allowing correct innervation of target organs and *Meis1* inactivation lead to altered retrograde transport^7^. Similar mechanisms may apply to other neuronal types and thus contribute to RLS and PLMS, opening new hypotheses about its participation in the underlying deleterious cellular mechanisms.

The present study has some limitations. Our RLS population was well-characterized but relatively small with demographic differences, *i.e.* age and gender, compared to healthy controls. This may explain why despite substantial differences in autonomic balance between patients and controls, some parameters failed to reach statistical significance, especially when these parameters were adjusted to gender and age. The sample size of RLS patients was limited by the difficulty in combining stringent selection criteria, mainly available drug-free PSG, PLMS above 15 per hour, and *MEIS1* SNP rs2300478 GG or TT homozygous. The two patient groups were deliberately chosen as extreme situations to investigate the involvement of *MEIS1* SNP in the sympathovagal balance during sleep in RLS patients and therefore patients heterozygote for rs2300478 were not included. With a frequency of about 4% for the GG genotype in the general population, we were unable to isolate any healthy homozygote subjects which prevented analysis of a potential effect on sympatho-vagal balance in normal controls. All patients included in the study were drug-free for at least 15 days prior to sleep recording; however 45% of them were previously exposed to a RLS treatment that may thus be considered as a limitation. Finally, to avoid artifacts, only ECG of PLM-free periods were analyzed that may have underestimated the significance of our results.

Another limitation relates to the interpretation with HRV analysis being an indirect assessment of the cardiac autonomic activity using noninvasive monitoring of electrocardiogram. The interpretation of HRV index is based on correlation between HRV changes and attempted cardiac autonomic regulation following experimental maneuvers, development of pathologies or pharmacological study^37^. It is now well admitted that the efferent vagal activity remains the major contributor to the HF component, also named respiratory band, as seen in both clinical and experimental observations of autonomic maneuvers such as electrical vagal stimulation, muscarinic receptor blockade, and vagotomy^46^,^47^,^48^. However, measurement of HRV under control breathing is a critical issue for ECG recordings^49^. In our study, we did not verified that respiratory frequency is within the HF band, and therefore cannot exclude that very low respiratory rate in particular impacts both total and HF power densities. In addition, the interpretation of the LF band and its relation to cardiac sympathetic activity is also controversial and still under debate^48^,^50^. In this work, interpretation of the results was mainly done using the classical recommendations for the standards of measurement, physiological interpretation, and clinical use of HRV for both practice and research^37^. Recent guidelines for psychiatry research preconize the same methodology for analysis and interpretation of HRV^51^. We also used Poincare plot geometry method to improve the analyses and interpretation of HRV measures as recently suggested^36^. However, both time domain and Poincare plot analyses were performed on short sequences *i.e.* 180 s which is below the recommended duration of 256 s. Briefly, since variance is mathematically the total power of spectral analysis, SDNN reflects cyclic components responsible for variability in the period of recording. When the monitored sequence is reduced in time, SDNN estimates shorter and shorter cycle lengths. Thus, the shorter is the analyzed ECG sequence, the more is the high frequency and SDNN arbitrarily increased^52^. This dependency of SDNN values on the length of the analyzed ECG sequences implies that on arbitrarily selected short ECGs, SDNN is a less reliable statistical parameter, should be cautiously interpreted and could explain some discrepancies found in the present study between time and frequency domain values in HRV.

Altogether our results demonstrate that RLS patients should be considered differently depending on their *MEIS1* genotype. The lack of parasympathetic activity during slow-wave sleep in RLS patients homozygous for the *MEIS1* risk allele may have clinical significance with potential links to the cardio-vascular risk observed in some patients, thus opening new perspectives for a personalized medical approach.

## Methods

### Population

Sixty-four drug-free patients (30 males and 34 females, mean age 58.9 ± 12.5) with primary RLS were included since 2010. Patients underwent a semi-structured assessment to confirm the RLS diagnosis using standard criteria^53^, and to detail the age of RLS onset, ferritin levels, presence of RLS symptoms in the arms, and family history of RLS. We excluded the presence of comorbidity suggestive of secondary RLS: iron deficiency, pregnancy, chronic renal failure, hemochromatosis or neurological diseases (Parkinson’s disease, multiple sclerosis, polyneuropathy, fibromyalgia, dementia, myelitis, spin cerebellar ataxia and narcolepsy). The RLS severity index was assessed according to the criteria of the International Restless Legs Syndrome Study Group (IRLSSG)^54^. Although diagnosed before 2014, these patients fulfilled the revised diagnostic criteria^55^. Nine out of 64 RLS patients were diagnosed with hypertension (systolic blood pressure ≥140 mmHg or diastolic blood pressure ≥90 mmHg) with 3 out of 20 in the RLS GG group and 6 out of 44 in the RLS TT group, and eight received a treatment with antihypertensive drugs at the time of study. Hypertensive patient treated with β-blocker were excluded.

As controls, 38 subjects (9 males and 29 females, mean age 44.9 ± 13.1) participated in the study. All controls had normal neurological examination, normal and regular sleep, no sleep complaint, and no RLS. Two control subjects had a treatment with antihypertensive drugs but no β-blocker.

This study was approved by the institutional review board of the University of Montpellier, France. Each participant signed legal informed consent forms. All methods were performed in accordance with the relevant guidelines and regulations.

### Genotyping

All RLS patients were genotyped for rs2300478 in the *MEIS1* locus as previously described^5^ and subdivided in two subgroups: one group homozygote G/G (at risk for RLS) and one group T/T (protective of RLS). RLS patients heterozygote for rs2300478 were not included.

### Polysomnography

All participants underwent one night of PSG recording in the sleep laboratory as previously described^56^. Twenty-nine patients (45%) were previously treated for RLS [dopaminergic agonists (ropinirole, pramipexole, rotigotine in 86%), alpha 2 delta-ligands (pregabaline, gabapentin, 7%), levodopa-benserazide (3%), clonazepam and opioids (codeine, 4%)]. However, none of the participants was taking central nervous system (dopaminergic agonists, levodopa, α2δ ligands, clonazepam and opioids) or any other medications known to influence sleep or movements at least 2 weeks prior to sleep recording.

All PSGs were scored manually for sleep stages, PLMS in NREM and REM sleep, and respiratory events according to standard criteria^57^. Only patients with PLMS index above 15 per hour were included. Participants with an index of apneas + hypopneas >15/hour were excluded.

### Heart rate variability analysis

HRV parameters were calculated from ECG (Electrocardiogram) taken during PSGs in period free of apneas, hypopneas and PLMS to avoid interferences caused by autonomic changes due to respiratory events and movements. HRV analyses were conducted during wakefulness before and after sleep onset, light NREM sleep (N2), slow-wave sleep (N3) and REM sleep stages. Only the data acquired in N2, N3 and REM sleep are presented. HRV analysis was performed using Kubios HRV analysis software^58^ according to international recommendations^37^. For each patient, 3 stationary ECG sequences of 3 minutes epoch duration without noise and missing data, artifact, conduction disturbance and cardiac ectopic beat were analyzed in each sleep stage. Each ECG signal was analyzed for automatic detection of R waves and beat-to-beat RR intervals were calculated. HRV was assessed using frequency and time domain analyses^37^. Data were sampled at 200 Hz. The interpolated R-R interval tachograms were then processed by the non-parametric Fast-Fourier Transform algorithm using Welch’s periodogram method (256 s length, overlapped by 50%). The low frequency power (LF range, 0.04–0.15 Hz)) and the high frequency power (HF range, 0.15–0.4 Hz) were calculated^37^. The efferent vagal activity is the major contributor to the HF component, including respiratory effect^59^, while the LF band reflects both baroreflex and sympathetic component of the autonomic nervous system^50^,^59^,^60^. The LF/HF ratio was calculated as an expression of the sympathovagal activity^37^,^51^. Linear time-domain measurements defined as the mean normal-to-normal R-R length (RRmean), standard deviation of normal-to-normal R-R intervals (SDNN), and root mean square of successive differences (RMSSD) were also performed on the same 3 min ECG periods. Results obtained from Poincare plot geometry analysis applied to HRV assessed the standard deviation of instantaneous beat-to-beat interval variability (SD1) and the standard deviation of continuous long-term R/R interval variability (SD2). HRV analysis was performed blinded to diagnosis and genotyping.

### Statistical analysis

Univariate analysis was performed for each variable. Data are presented as Median ± Median Absolute Deviation (MAD) in tables and as lower quartile, median and upper quartile (boxes), and minimum and maximum ranges (whiskers) in figures, and categorical variables as numbers and percentages. We used χ^2^ test for categorical variables and Mann-Whitney or Student *t* test for quantitative variables, according to the normality of the distribution, assessed with the Shapiro-Wilk test.

As control and RLS groups differed in age and sex, statistical comparison between both groups was conducted using a statistical model adjusted on these two parameters. Generalized linear mixed-effects model for repeated measures was used to take into account repeated measures. Similar comparisons were made in RLS patients according to the *MEIS1* genotype. These statistical analyses were performed using R version 2.15.2. For all analyses, the level of significance was set at p ≤ 0.05.

## Additional Information

**How to cite this article:** Thireau, J. *et al*. *MEIS1* variant as a determinant of autonomic imbalance in Restless Legs Syndrome. *Sci. Rep.* **7**, 46620; doi: 10.1038/srep46620 (2017).

**Publisher's note:** Springer Nature remains neutral with regard to jurisdictional claims in published maps and institutional affiliations.

## Supplementary Material

Supplementary Information

## Figures and Tables

**Table 1:** Clinical, biological and polysomnographical characteristics of RLS patients, healthy controls, and RLS patients sorted according to the *MEIS1* SNP rs2300478 genotype (GG or TT).

	Control	RLS	*p*	*p*_*a*_	RLS GG	RLS TT	*p*
N	38	64	NA	NA	20	44	NA
Age	45.0 ± 9.5	61.5 ± 8.8	5.10^−7^***	NA	67.0 ± 8.0	60.0 ± 6.1	0.57
Men, n (%)	9 (23.7%)	30 (46.9%)	0.03*	NA	10 (50%)	20 (45.5%)	0.95
BMI	26.9 ± 5.2	25.9 ± 3.1	0.38	0.792	25.4 ± 2.5	26.8 ± 3.2	0.22
Ferritin	68.5 ± 42.5	83.0 ± 50.0	0.12	0.911	84.5 ± 52.0	80.0 ± 48.5	0.76
Age onset	NA	45.0 ± 12.5	NA	NA	38.0 ± 13.0	50.0 ± 8.0	0.44
RLS sev.	NA	25.0 ± 4.0	NA	NA	26.5 ± 5.5	24.0 ± 5.0	0.28
TST (min)	382 ± 41	346 ± 48	0.0007***	0.0046^##^	350 ± 67	346 ± 47	0.62
SE (%)	85 ± 8	74 ± 9	0.0001***	0.0748	70 ± 12	75 ± 9	0.79
WASO	36 ± 18	88 ± 31	0.0003***	0.0909	83 ± 19	89 ± 45	0.42
SL	16 ± 12	19 ± 11	0.45	0.623	21 ± 13	19 ± 10	0.17
N1 (%)	6.1 ± 3.1	7.8 ± 2.7	0.026*	0.4104	7.5 ± 2.6	8.8 ± 6.3	0.44
N2 (%)	54.0 ± 3.5	49.8 ± 5.0	0.034*	0.00265^##^	47.9 ± 6.7	50.2 ± 8.4	0.81
N3 (%)	21.0 ± 3.2	21.1 ± 6.4	0.98	0.16679	20.9 ± 7.3	22.2 ± 8.4	0.45
REM (%)	16.7 ± 4.0	19.0 ± 4.3	0.35	0.0572	19.4 ± 4.7	18.9 ± 6.5	0.41
iPLM_NREM_	1.1 ± 1.1	38.3 ± 20.6	1.10^−4^***	0.00042^###^	62.6 ± 36.2	29.9 ± 15.8	4.10^−9^***
iPLM_REM_	0 ± 0	4.1 ± 4.1	8.10^−9^***	0.08718	12.4 ± 11.6	2.2 ± 2.2	0.32
AHI	1.9 ± 1.4	4.4 ± 4.1	0.19	0.7298	5.2 ± 5.0	4.4 ± 3.9	0.49
Min SaO_2_	91.5 ± 2.5	88.0 ± 4.0	0.16	0.8966	87.5 ± 3.5	88.0 ± 4.0	0.71
Mean SaO_2_	95.5 ± 1.5	95.0 ± 1.0	0.41	0.173	94.0 ± 2.0	95.0 ± 1.0	0.64
tSA O_2_ <90%	0 ± 0	0.11 ± 0.11	0.19	0.3758	0.17 ± 0.17	0.04 ± 0.04	0.38

BMI = body mass index; Ferritin = ferritin blood levels, RLS sev. = RLS severity index; TST = Total Sleep Time in minutes; SE = Sleep Efficiency; WASO = Wake After Sleep Onset; SL = Sleep Latency; N1 (%) = percentage of cumulated N1 stages duration over TST; N2 (%) = percentage of cumulated N2 stages duration over TST; N3 (%) = percentage of cumulated N3 stages duration over TST and REM (%) = percentage of cumulated REM stages duration over TST; iPLM_NREM_ = PLM index during NREM sleep; iPLM_REM_ = PLM index during REM sleep; AHI = apnea/hypopnea per hour of sleep index; Min SaO_2_ = minimal oxygen saturation; Mean SaO_2_ = average of oxygen saturation; tSA O_2_ <90% = time spent with an oxygen saturation inferior to 90% of total sleeping time. NA = Not Applicable. Data are presented as Median ± MAD. *p* = p value following Student or Wilcoxon’s test; *p*_*a*_ = p value adjusted for age and gender. *p ≤ 0.05; ***p ≤ 0.001.

**Table 2:** Results of heart rate variability analysis in the frequency domain in the different sleep stages in RLS patients and healthy controls, and in RLS patients sorted according to the *MEIS1* SNP rs2300478 genotype (GG or TT).

			N2	N3	REM
Control	LF	Median ± MAD	35.7 ± 11.6	35.4 ± 10.0	29.5 ± 8.5
	HF	Median ± MAD	25 ± 9.15	36.1 ± 15.7	18.9 ± 7.3
	LF/HF	Median ± MAD	1.81 ± 0.85	1.18 ± 0.71	1.82 ± 0.77
RLS	LF	Median ± MAD	43.3 ± 16.3	37.1 ± 10.8	27.2 ± 6.8
	HF	Median ± MAD	19.7 ± 10.3	25.7 ± 13.0	14.6 ± 7.2
	LF/HF	Median ± MAD	2.43 ± 1.42	1.47 ± 0.71	1.79 ± 0.77
Non adjusted	LF	*p*	0.002***	0.44	0.52
	HF	*p*	0.07	0.015*	0.57
	LF/HF	*p*	0.004***	0.17	0.09
Adjusted	LF	*p*_*a*_	0.059	0.71	0.64
	HF	*p*_*a*_	0.81	0.57	0.8
	LF/HF	*p*_*a*_	0.09	0.58	0.12
RLS TT	LF	Median ± MAD	39.9 ± 11.9	36.7 ± 11.9	28.1 ± 6.2
	HF	Median ± MAD	21.9 ± 12.1	27.3 ± 10.4	16.4 ± 8.7
	LF/HF	Median ± MAD	1.94 ± 0.95	1.35 ± 0.68	1.69 ± 0.65
RLS GG	LF	Median ± MAD	59.3 ± 15.8	38.6 ± 8.0	26.9 ± 2.5
	HF	Median ± MAD	14.4 ± 7.8	13.2 ± 10.0	13.2 ± 4.9
	LF/HF	Median ± MAD	4.00 ± 2.41	1.99 ± 1.09	1.88 ± 1.05
	LF	*p*	0.13	0.78	0.43
	HF	*p*	0.18	0.02*	0.09
	LF/HF	*p*	0.18	0.05*	0.13

LF = Low Frequency; HF = High Frequency. Data are presented as Median ± MAD. *p* = p value following Student or Wilcoxon’s test; *p*_*a*_ = p value adjusted for age and gender, or PLMS. *p ≤ 0.05; ***p ≤ 0.005.
