# Supplementary material for: MEIS1 variant as a determinant of autonomic imbalance in Restless Legs Syndrome
Source: Sci Rep. 2017 Apr 20;7:46620. doi: 10.1038/srep46620 (PMC5397858; doi:10.1038/srep46620)
Supplement: Supplementary Information [file srep46620-s1.pdf]

MEIS1 variant as a determinant of autonomic imbalance in Restless Legs Syndrome

Authors: Jérôme Thireau, PhD[1§], Charlotte Farah, PhD[1§], Nicolas Molinari, PhD[2], Fabrice Bouilloux, PhD[5], Lucas Torreilles[1], Juliane Winkelmann, MD, PhD[3], Sabine Scholz[4], Sylvain Richard, PhD[1], Yves Dauvilliers, MD, PhD[4§]* and Frédéric Marmigère, PhD[5§]*.

1- INSERM U1046, UMR CNRS 9214, University of Montpellier, 371, avenue du doyen Gaston Giraud, 34295 Montpellier Cedex 05, France.
2- IMAG U519, University of Montpellier, Department of statistics, La Colombière hospital, Montpellier, France.
3- Institute for Neurogenomic, Helmholtz Zentrum München, Neuherberg; Neurologische Klinik und Poliklinik Munich, Germany.
4- Sleep Unit, Department of Neurology, Gui-de-Chauliac hospital, INSERM U1061, Montpellier, France.
5- INSERM U1051, Institute for Neurosciences of Montpellier (INM), 80 rue Augustin Fliche, 34091 Montpellier, France.

*Corresponding authors: frederic.marmigere@inserm.fr; y-dauvilliers@chu-montpellier.fr
§ Equal contribution

Running title: Heart rate variability in RLS patients

Keywords: RLS, MEIS1, Periodic Limb Movement, sleep, sympathetic, parasympathetic, heart rate variability

Supplementary figures and tables:

Supplementary Figure S1: Box-whisker plots showing RRmean, SDNN and RMSSD in control subjects (a), RLS patients (b), RLS TT patients (c) and RLS GG patients (d) during N2, N3 and REM sleep stages. Data are represented as lower quartile, median and upper quartile (boxes), and minimum and maximum ranges (whiskers). Red dashed lines indicate the median values for RRmean, SDNN and RMSSD in control patients during N2. * $p \leq 0.05$; ** $p \leq 0.01$; *** $p \leq 0.005$ following Student's or Wilcoxon's tests. $^{\#}$ $p_a \leq 0.05$; $^{\#\#\#}$ $p_a \leq 0.005$ following statistical test adjusted for age and gender (Control *vs* RLS) or for PLMS (RLS TT *vs* RLS GG). RRmean= mean normal-to-normal R-R length; SDNN=standard deviation of normal-to-normal R-R intervals; RMSSD=root mean square of successive differences.

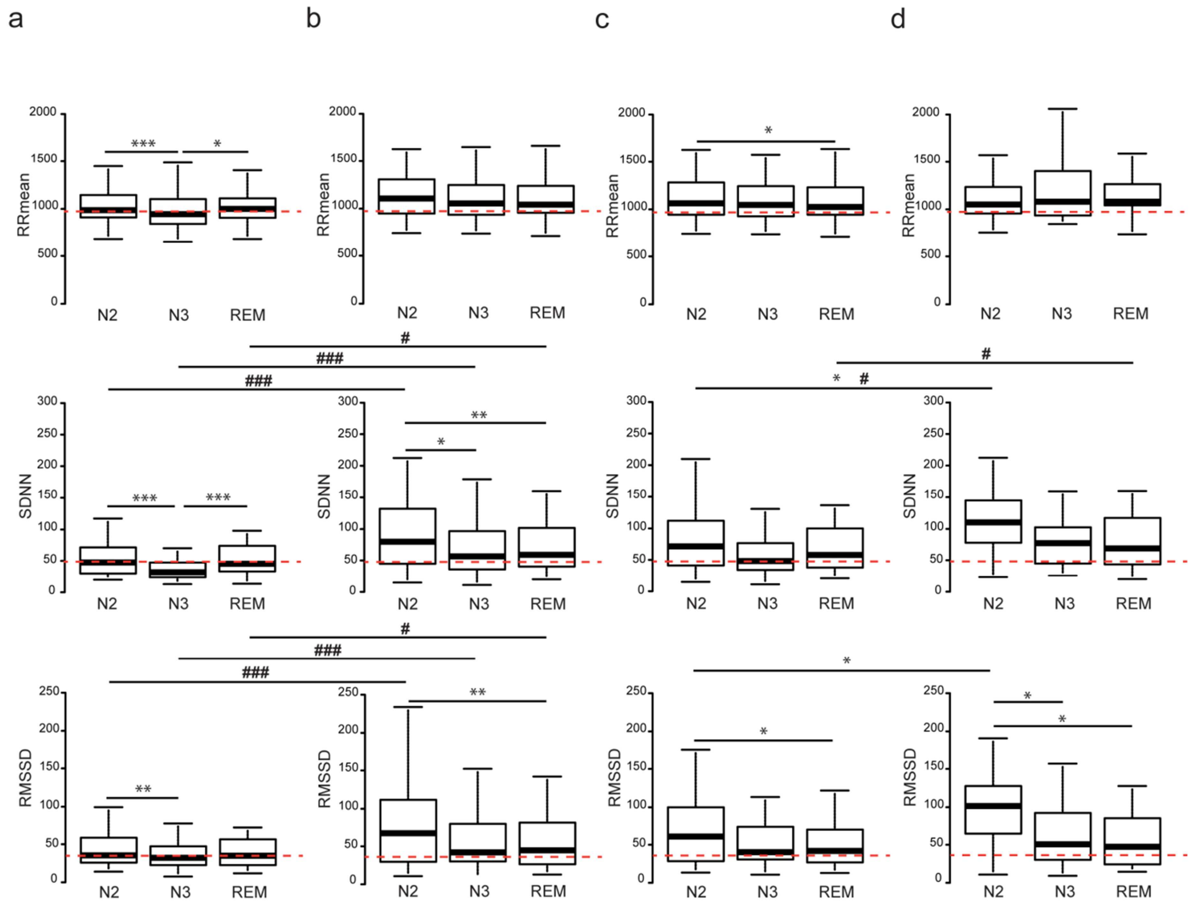

Supplementary Figure S2: Box-whisker plots showing SD1 and SD2 in control subjects (a), RLS patients (b), RLS TT patients (c) and RLS GG patients (d) during N2, N3 and REM sleep stages. Data are represented as lower quartile, median and upper quartile (boxes), and minimum and maximum ranges (whiskers). Red dashed lines indicate the median values for SD1 or SD2 in control patients during N2. * $p \le 0.05$; ** $p \le 0.01$; *** $p \le 0.005$ following Student's or Wilcoxon's tests. [#] $p_a \le 0.05$; [###] $p_a \le 0.005$ following statistical test adjusted for age and gender (Control *vs* RLS) or for PLMS (RLS TT *vs* RLS GG). SD1=standard deviation of instantaneous beat-to-beat interval variability; SD2=standard deviation of continuous long-term R/R interval variability.

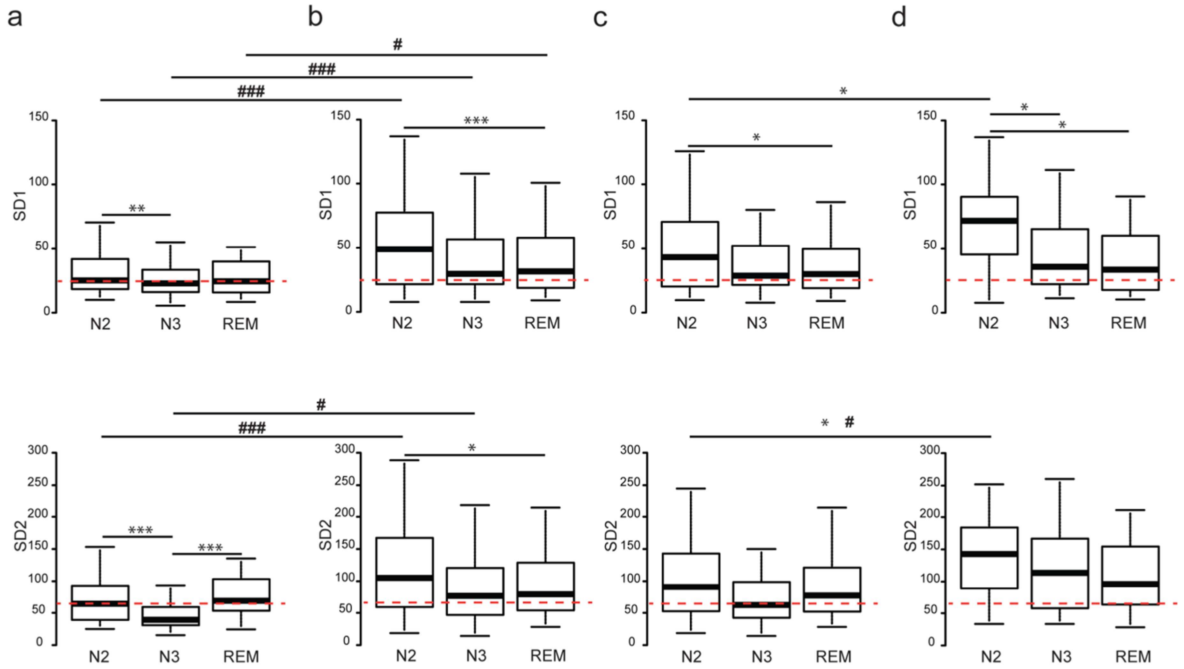

Supplementary Table S1: Results of heart rate variability analysis in the time-domain in the different sleep stages in RLS patients and healthy controls, and in RLS patients sorted according to the *MEIS1* SNP rs2300478 genotype (GG or TT). Data are presented as Median ± MAD. p=p value following Student or Wilcoxon's test; p_a= p value adjusted for age and gender. * $p \leq 0.05$; ** $p \leq 0.01$ *** $p \leq 0.005$. RRmean= mean normal-to-normal R-R length; SDNN=standard deviation of normal-to-normal R-R intervals; RMSSD=root mean square of successive differences.

			N2	N3	REM
**Control**	**Mean RR**	**Median**±MAD	**988.3**±123.9	**945.9**±155.8	**1000.1**±109.2
	**SDNN**	**Median**±MAD	**47.6**±19.5	**32.3**±10.4	**45.8**±19.7
	**RMSSD**	**Median**±MAD	**35.5**±16.3	**32.0**±10.5	**34.7**±18.2
**RLS**	**Mean RR**	**Median**±MAD	**1060.6**±172.4	**1057.0**±161.0	**1042.1**±123.8
	**SDNN**	**Median**±MAD	**83.6**±42.4	**56.7**±24.0	**58.9**±25.5
	**RMSSD**	**Median**±MAD	**68.6**±38.6	**41.6**±19.2	**44.6**±21.4
**Non adjusted**	**Mean RR**	p	**0.034***	**0.003*****	**0.042***
	**SDNN**	p	**0.0003*****	**2.10^{-5}*****	**0.047***
	**RMSSD**	p	**6.10^{-4}*****	**9.10^{-4}*****	**0.035***
**Adjusted**	**Mean RR**	p_a	**0.36**	**0.12**	**0.40**
	**SDNN**	p_a	**0.003*****	**4.10^{-4}*****	**0.03***
	**RMSSD**	p_a	**2.10^{-4}*****	**0.0026*****	**0.017***
**RLS TT**	**Mean RR**	**Median**±MAD	**1062.7**±176.0	**1048.3**±158.9	**1026.3**±159.7
	**SDNN**	**Median**±MAD	**71.5**±35.3	**48.2**±19.5	**58.0**±24.6
	**RMSSD**	**Median**±MAD	**60.7**±35.2	**40.3**±15.8	**42.0**±18.8
**RLS GG**	**Mean RR**	**Median**±MAD	**1049.9**±117.8	**1081.5**±155.9	**1082.3**±90.2
	**SDNN**	**Median**±MAD	**109.9**±37.4	**77.0**±28.8	**68.9**±26.4
	**RMSSD**	**Median**±MAD	**101.1**±29.8	**50.3**±27.6	**47.1**±24.8
	**Mean RR**	p	**0.72**	**0.20**	**0.22**
	**SDNN**	p	**0.025***	**0.06**	**0.42**
	**RMSSD**	p	**0.02***	**0.78**	**0.59**
	**Mean RR**	p_a	**0.83**	**0.38**	**0.26**
	**SDNN**	p_a	**0.024***	**0.58**	**0.038***
	**RMSSD**	p_a	**0.056**	**0.48**	**0.39**

Supplementary Table S2: Poincare plot geometry analysis of heart rate variability in the different sleep stages in RLS patients and healthy controls, and in RLS patients sorted according to the *MEIS1* SNP rs2300478 genotype (GG or TT). Data are presented as Median ± MAD. *p*=p value following Student or Wilcoxon's test; p_a= p value adjusted for age and gender. * $p \leq 0.05$; ** $p \leq 0.01$ *** $p \leq 0.005$. SD1=standard deviation of instantaneous beat-to-beat interval variability; SD2=standard deviation of continuous long-term R/R interval variability.

			**N2**	**N3**	**REM**
**Control**	**SD1**	**Median**±MAD	**25.2**±11.6	**22.7**±7.4	**24.6**±12.9
	**SD2**	**Median**±MAD	**64.3**±25.8	**39.4**±12.4	**69.1**±24.5
**RLS**	**SD1**	**Median**±MAD	**48.9**±27.5	**29.5**±13.6	**31.6**±15.2
	**SD2**	**Median**±MAD	**105.1**±52.8	**76.3**±33.6	**79.4**±31.5
**Non adjusted**	**SD1**	***p***	**6.10^{-4}*****	**0.002*****	**0.036***
	**SD2**	***p***	**4.10^{-4}*****	**0.012***	**0.080**
**Adjusted**	**SD1**	**p_a**	**2.10^{-4}*****	**4.10^{-3}*****	**0.017***
	**SD2**	**p_a**	**8.10^{-3}*****	**0.019***	**0.08**
**RLS TT**	**SD1**	**Median**±MAD	**43.0**±25.2	**28.6**±11.2	**29.9**±13.5
	**SD2**	**Median**±MAD	**90.4**±41.9	**62.4**±27.0	**77.2**±30.1
**RLS GG**	**SD1**	**Median**±MAD	**71.6**±21.2	**35.7**±18.5	**33.5**±17.6
	**SD2**	**Median**±MAD	**142.7**±48.8	**113.3**±54.4	**95.9**±33.6
	**SD1**	***p***	**0.021***	**0.099**	**0.59**
	**SD2**	***p***	**0.036***	**0.50**	**0.44**
	**SD1**	**p_a**	**0.057**	**0.44**	**0.39**
	**SD2**	**p_a**	**0.028***	**0.94**	**0.37**
